# Supplementary material for: Standardization of Somatic Variant Classifications in Solid and Haematological Tumours by a Two-Level Approach of Biological and Clinical Classes: An Initiative of the Belgian ComPerMed Expert Panel
Source: Cancers (Basel). 2019 Dec 16;11(12):2030. doi: 10.3390/cancers11122030 (PMC6966529; doi:10.3390/cancers11122030)
Supplement: Supplementary file 1 [file cancers-11-02030-s001.pdf]

cancers

MDPI

Supplementary Materials

Table 1. Classification of the most commonly mutated cancer genes as Tumor suppressor gene (TsG) or Oncogene based on 4 sources: OncoKb, Vanderbilt TSGene 2.0, Vogelstein et al 2013, and IntOGen.

HugoSymbol	OncoKb 2019	Vanderbilt 2019	Vogelstein 2013	IntOGen 2019	Merge
ABL1	Oncogene	Oncogene	Oncogene	Oncogene	Oncogene
ACVR1	Oncogene				Oncogene
ACVR1B			TsG		TsG
AKT1	Oncogene	Oncogene	Oncogene	Oncogene	Oncogene
AKT2	Oncogene	Oncogene			Oncogene
AKT3	Oncogene				Oncogene
ALK	Oncogene	Oncogene	Oncogene	Oncogene	Oncogene
AMER1	TsG			TsG	TsG
ANKRD11	TsG				TsG
APC	TsG	TsG	TsG	TsG	TsG
AR	Oncogene	Oncogene	Oncogene	Oncogene	Oncogene
ARAF	Oncogene	Oncogene		Oncogene	Oncogene
ARID1A	TsG	TsG	TsG	TsG	TsG
ARID1B	TsG		TsG	TsG	TsG
ARID2	TsG	TsG	TsG	TsG	TsG
ARID5B	TsG				TsG
ASXL1	TsG	TsG	TsG	TsG	TsG
ASXL2	TsG			TsG	TsG
ATM	TsG	TsG	TsG	TsG	TsG
ATR	TsG	TsG			TsG
ATRX	TsG		TsG	TsG	TsG
AURKA	Oncogene	Oncogene			Oncogene
AURKB	Oncogene				Oncogene
AXIN1	TsG	TsG	TsG	TsG	TsG
AXIN2	TsG	TsG		TsG	TsG
AXL	Oncogene	Oncogene			Oncogene
B2M	TsG		TsG	TsG	TsG
BAP1	TsG	TsG	TsG	TsG	TsG
BARD1	TsG	TsG			TsG
BCL10	TsG	TsG			TsG
BCL2	Oncogene	Oncogene	Oncogene	Oncogene	Oncogene
BCL2L11	TsG				TsG
BCL6	Oncogene	Oncogene		Oncogene	Oncogene
BCOR	TsG		TsG	TsG	TsG
BLM	TsG	TsG			TsG
BRAF	Oncogene	Oncogene	Oncogene	Oncogene	Oncogene
BRCA1	TsG	TsG	TsG	TsG	TsG
BRCA2	TsG	TsG	TsG	TsG	TsG
BRD4	Oncogene			Oncogene	Oncogene
BTK	Oncogene			Oncogene	Oncogene
CALR	Oncogene				Oncogene
CARD11	Oncogene	Oncogene	Oncogene	Oncogene	Oncogene
CASP8	TsG	TsG	TsG	TsG	TsG
CBL	TsG	Oncogene	Oncogene	Oncogene	Oncogene/TsG
CCND1	Oncogene	Oncogene		Oncogene	Oncogene
CCND2	Oncogene			Oncogene	Oncogene
CCND3	Oncogene			Oncogene	Oncogene
CCNE1	Oncogene				Oncogene
CDC73	TsG	TsG	TsG	TsG	TsG
CDH1	TsG	TsG	TsG	TsG	TsG

CDK4	Oncogene			Oncogene	Oncogene
CDK6	Oncogene				Oncogene
CDKN1A	TsG	TsG		TsG	TsG
CDKN1B	TsG	Oncogene		TsG	Oncogene/TsG
CDKN2A	TsG	TsG	TsG	TsG	TsG
CDKN2B	TsG	TsG			TsG
CDKN2C	TsG	TsG		TsG	TsG
CEBPA	TsG	TsG	TsG		TsG
CHEK1	TsG	TsG			TsG
CHEK2	TsG	TsG		TsG	TsG
CIC	TsG		TsG	TsG	TsG
CREBBP	TsG	TsG	TsG	TsG	TsG
CRKL	Oncogene				Oncogene
CRLF2	Oncogene	Oncogene	Oncogene		Oncogene
CSF1R		Oncogene	Oncogene		Oncogene
CSF3R	Oncogene			Oncogene	Oncogene
CTCF	TsG	TsG		TsG	TsG
CTLA4	Oncogene				Oncogene
CTNNB1	Oncogene	Oncogene	Oncogene	Oncogene	Oncogene
CYLD	TsG	TsG	TsG	TsG	TsG
DAXX	TsG		TsG	TsG	TsG
DDR2	Oncogene				Oncogene
DNMT1	Oncogene	Oncogene	Oncogene		Oncogene
DNMT3A	TsG	Oncogene	Oncogene	TsG	Oncogene/TsG
E2F3	Oncogene				Oncogene
EGFR	Oncogene	Oncogene	Oncogene	Oncogene	Oncogene
EP300	TsG		TsG	TsG	TsG
ERBB2	Oncogene	Oncogene	Oncogene	Oncogene	Oncogene
ERBB3	Oncogene			Oncogene	Oncogene
ERBB4	Oncogene	TsG		TsG	Oncogene/TsG
ERCC2	TsG			TsG	TsG
ERCC3	TsG			TsG	TsG
ERCC4	TsG				TsG
ERF	TsG	TsG			TsG
ERG	Oncogene	Oncogene		Oncogene	Oncogene
ERRFI1	TsG	TsG			TsG
ESR1	Oncogene			Oncogene	Oncogene
ETV1	Oncogene	Oncogene			Oncogene
ETV6	TsG	Oncogene/TsG		Oncogene	Oncogene/TsG
EWSR1	Oncogene	Oncogene		Oncogene	Oncogene
EZH2	Oncogene/TsG	Oncogene	Oncogene	TsG	Oncogene/TsG
FAM123B		TsG	TsG		TsG
FANCA	TsG			TsG	TsG
FANCC	TsG				TsG
FAT1	TsG	TsG		TsG	TsG
FBXW7	TsG	TsG	TsG	TsG	TsG
FGF19	Oncogene				Oncogene
FGF3	Oncogene	Oncogene			Oncogene
FGF4	Oncogene	Oncogene			Oncogene
FGFR1	Oncogene			Oncogene	Oncogene
FGFR2	Oncogene	Oncogene	Oncogene	Oncogene	Oncogene
FGFR3	Oncogene	Oncogene	Oncogene	Oncogene	Oncogene
FGFR4	Oncogene			Oncogene	Oncogene
FH	TsG	TsG			TsG
FLT1	Oncogene				Oncogene
FLT3	Oncogene	Oncogene	Oncogene	Oncogene	Oncogene
FLT4	Oncogene				Oncogene
FOXA1	Oncogene/TsG			TsG	Oncogene/TsG

FOXL2	Oncogene/TsG	Oncogene	Oncogene	Oncogene	Oncogene/TsG
FUBP1	TsG		TsG	TsG	TsG
GATA1			TsG	Oncogene	Oncogene/TsG
GATA2	Oncogene	Oncogene	Oncogene	Oncogene	Oncogene
GATA3	Oncogene/TsG		TsG	TsG	Oncogene/TsG
GLI1	Oncogene	Oncogene		Oncogene	Oncogene
GNA11	Oncogene	Oncogene	Oncogene	Oncogene	Oncogene
GNAQ	Oncogene	Oncogene	Oncogene	Oncogene	Oncogene
GNAS	Oncogene	Oncogene	Oncogene	Oncogene	Oncogene
H3F3A	Oncogene	Oncogene	Oncogene	Oncogene	Oncogene
HGF	Oncogene				Oncogene
HIST1H3B		Oncogene	Oncogene	Oncogene	Oncogene
HLA-A	TsG				TsG
HLA-B	TsG				TsG
HNF1A	TsG		TsG	TsG	TsG
HRAS	Oncogene	Oncogene	Oncogene	Oncogene	Oncogene
IDH1	Oncogene	Oncogene/TsG	Oncogene	Oncogene	Oncogene/TsG
IDH2	Oncogene	Oncogene	Oncogene	Oncogene	Oncogene
IGF1	Oncogene				Oncogene
IGF1R	Oncogene				Oncogene
IGF2	Oncogene				Oncogene
INPP4B	TsG	TsG			TsG
IRS2	Oncogene				Oncogene
JAK1	Oncogene/TsG	Oncogene	Oncogene		Oncogene/TsG
JAK2	Oncogene	Oncogene	Oncogene		Oncogene
JAK3	Oncogene	Oncogene	Oncogene	Oncogene	Oncogene
JUN	Oncogene	Oncogene			Oncogene
KDM5C	TsG		TsG	TsG	TsG
KDM6A	TsG		TsG	TsG	TsG
KDR	Oncogene			Oncogene	Oncogene
KEAP1	TsG			Oncogene	Oncogene/TsG
KIT	Oncogene	Oncogene	Oncogene	Oncogene	Oncogene
KLF4	Oncogene/TsG	Oncogene/TsG	Oncogene	Oncogene	Oncogene/TsG
KMT2A	TsG	Oncogene		TsG	Oncogene/TsG
KMT2B	TsG			TsG	TsG
KMT2C	TsG			TsG	TsG
KMT2D	TsG			TsG	TsG
KRAS	Oncogene	Oncogene	Oncogene	Oncogene	Oncogene
MAP2K1	Oncogene	Oncogene	Oncogene	Oncogene	Oncogene
MAP2K4	TsG	TsG		TsG	TsG
MAP3K1	TsG		TsG	TsG	TsG
MAPK1	Oncogene			Oncogene	Oncogene
MDM2	Oncogene	Oncogene		Oncogene	Oncogene
MDM4	Oncogene				Oncogene
MED12	Oncogene/TsG	Oncogene	Oncogene	Oncogene	Oncogene/TsG
MEF2B	Oncogene			TsG	Oncogene/TsG
MEN1	TsG	TsG	TsG	TsG	TsG
MET	Oncogene	Oncogene	Oncogene	Oncogene	Oncogene
MGA	TsG			TsG	TsG
MLH1	TsG	TsG	TsG		TsG
MLL2			TsG		TsG
MLL3			TsG		TsG
MPL	Oncogene	Oncogene	Oncogene		Oncogene
MSH2	TsG	TsG	TsG		TsG
MSH6	TsG		TsG		TsG
MTOR	Oncogene			Oncogene	Oncogene
MYC	Oncogene	Oncogene		Oncogene	Oncogene
MYCN	Oncogene	Oncogene		Oncogene	Oncogene

MYD88	Oncogene	Oncogene	Oncogene	Oncogene	Oncogene
NCOR1	TsG		TsG	TsG	TsG
NF1	TsG	TsG	TsG	TsG	TsG
NF2	TsG	TsG	TsG	TsG	TsG
NFE2L2	Oncogene	Oncogene	Oncogene	Oncogene	Oncogene
NFKBIA	TsG			TsG	Oncogene
NOTCH1	Oncogene/TsG	Oncogene/TsG	TsG	Oncogene/TsG	Oncogene/TsG
NOTCH2	Oncogene/TsG	Oncogene	TsG	Oncogene/TsG	Oncogene/TsG
NPM1	TsG	Oncogene	TsG	Oncogene/TsG	Oncogene/TsG
NRAS	Oncogene	Oncogene	Oncogene	Oncogene	Oncogene
NTRK1	Oncogene	Oncogene		Oncogene	Oncogene
NTRK2	Oncogene				Oncogene
NTRK3	Oncogene			Oncogene	Oncogene
PARP1	TsG				Oncogene
PAX5	TsG	Oncogene/TsG	TsG		Oncogene/TsG
PAX8	Oncogene				Oncogene
PBRM1	TsG	TsG	TsG	TsG	TsG
PDGFRA	Oncogene	Oncogene	Oncogene	Oncogene	Oncogene
PDGFRB	Oncogene	Oncogene		Oncogene	Oncogene
PHF6	TsG	TsG	TsG	TsG	TsG
PIK3CA	Oncogene	Oncogene	Oncogene	Oncogene	Oncogene
PIK3CB	Oncogene			Oncogene	Oncogene
PIK3CD	Oncogene				Oncogene
PIK3CG	Oncogene				Oncogene
PIK3R1	TsG		TsG	TsG	TsG
PIK3R3	TsG				TsG
PMS1	TsG	TsG			TsG
POLD1	TsG			Oncogene	Oncogene/TsG
POLE	TsG				TsG
PPM1D	Oncogene			TsG	Oncogene/TsG
PPP2R1A	TsG	Oncogene	Oncogene	Oncogene	Oncogene
PPP6C	TsG			TsG	TsG
PRDM1	TsG	TsG	TsG	TsG	TsG
PRKCI	Oncogene	Oncogene/TsG			Oncogene/TsG
PTCH1	TsG	TsG	TsG	TsG	TsG
PTEN	TsG	TsG	TsG	TsG	TsG
PTPN11	Oncogene	Oncogene	Oncogene	Oncogene	Oncogene
RAC1	Oncogene			Oncogene	Oncogene
RAD21	TsG			TsG	TsG
RAD50	TsG				TsG
RAD51	TsG				TsG
RAD51C	TsG	TsG			TsG
RAD51D	TsG				TsG
RAF1	Oncogene	Oncogene		Oncogene	Oncogene
RASA1	TsG	Oncogene		TsG	Oncogene/TsG
RB1	TsG	Oncogene/TsG	TsG	TsG	Oncogene/TsG
RBM10	TsG			TsG	TsG
RET	Oncogene	Oncogene	Oncogene	Oncogene	Oncogene
RHEB	Oncogene				Oncogene
RHOA	Oncogene	Oncogene/TsG		Oncogene	Oncogene/TsG
RICTOR	Oncogene				Oncogene
RIT1	Oncogene				Oncogene
RNF43	TsG		TsG	TsG	TsG
ROS1	Oncogene	Oncogene			Oncogene
RPTOR	Oncogene				Oncogene
RRAS2	Oncogene	Oncogene		Oncogene	Oncogene
RUNX1	TsG	Oncogene	TsG		TsG
RYBP	TsG				TsG

SDHA	TsG	TsG			TsG
SDHB	TsG	TsG			TsG
SDHC	TsG				TsG
SDHD	TsG	TsG			TsG
SETBP1	Oncogene	Oncogene	Oncogene	Oncogene	Oncogene
SETD2	TsG		TsG	TsG	TsG
SF3B1	Oncogene	Oncogene	Oncogene	Oncogene	Oncogene
SHQ1	TsG				TsG
SMAD2	TsG	TsG	TsG	TsG	TsG
SMAD3	TsG	TsG		TsG	TsG
SMAD4	TsG	TsG	TsG	TsG	TsG
SMARCA4	TsG	TsG	TsG	TsG	TsG
SMARCB1	TsG	TsG	TsG	TsG	TsG
SMO	Oncogene	Oncogene/TsG	Oncogene	Oncogene	Oncogene/TsG
SOCS1	TsG	TsG	TsG	TsG	TsG
SOX17	TsG			Oncogene	TsG
SOX9	Oncogene/TsG		TsG	TsG	Oncogene/TsG
SPEN	TsG				TsG
SPOP	TsG	Oncogene	Oncogene	Oncogene	Oncogene/TsG
SRC	Oncogene	Oncogene			Oncogene
SRSF2		Oncogene	Oncogene	Oncogene	Oncogene
STAG2	TsG		TsG	TsG	TsG
STAT3	Oncogene				Oncogene
STK11	TsG	TsG	TsG	TsG	TsG
TCF7L2	TsG	TsG		Oncogene	Oncogene/TsG
TERT	Oncogene				Oncogene
TET2	TsG	TsG	TsG	TsG	TsG
TGFBR1	TsG				TsG
TGFBR2	TsG	TsG		TsG	TsG
TMPRSS2					Oncogene
TNFAIP3	TsG	TsG	TsG	TsG	TsG
TOP1	TsG	Oncogene		TsG	Oncogene/TsG
TP53	TsG	TsG	TsG	TsG	TsG
TP53BP1	TsG				TsG
TRAF7			TsG		TsG
TSC1	TsG	TsG	TsG	TsG	TsG
TSC2	TsG	TsG		TsG	TsG
TSHR	Oncogene	Oncogene	Oncogene		Oncogene
U2AF1	Oncogene	Oncogene	Oncogene	Oncogene	Oncogene
VHL	TsG	TsG	TsG	TsG	TsG
WT1	Oncogene/TsG	Oncogene/TsG	TsG	TsG	Oncogene/TsG
XRCC2	TsG				TsG
YAP1	Oncogene	Oncogene/TsG			Oncogene/TsG

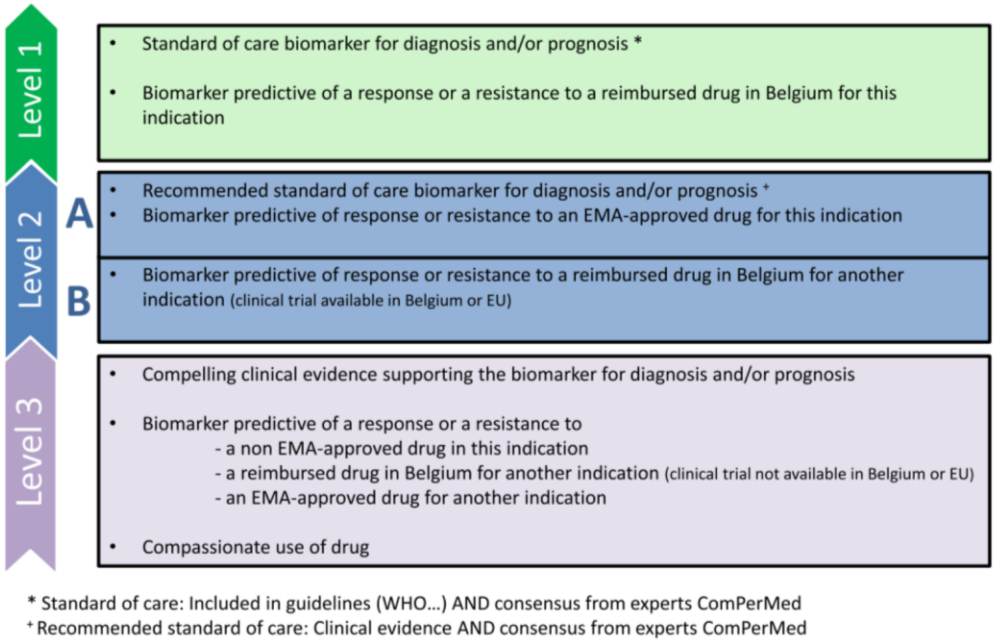

Figure S1. Definition of levels for diagnostic/prognostic or therapeutic biomarkers and molecular tests, according to the Belgian healthcare system. Taken with permission from Hèbrant et al., Belg. J. Med. Oncol. 2018 [1].

References

1 Hébrant, A.; Van Valckenborgh, E.; Salgado, R.; Froyen, G.; Hulstaert, F.; Roberfroid, D.; Tejpar, S.; Jouret-Mourin, A.; Van den Bulcke, M.; Waeytens, A. Opportunities and challenges in oncology and molecular testing: the Belgian strategy. *Belg. J. Med. Oncol.* 2018, *12*, 46–50.
